# STAT3 gain-of-function is not responsible for low total IgE levels in patients with autoimmune chronic spontaneous urticaria

**DOI:** 10.3389/fimmu.2022.902652

**Published:** 2022-07-19

**Authors:** Merle Sauer, Jörg Scheffel, Stefan Frischbutter, Niklas Mahnke, Marcus Maurer, Thomas Burmeister, Karoline Krause, Martin Metz

**Affiliations:** ^1^ Institute of Allergology, Charité - Universitätsmedizin Berlin, Berlin, Germany; ^2^ Fraunhofer Institute for Translational Medicine and Pharmacology (ITMP), Allergology and Immunology, Berlin, Germany; ^3^ Department of Hematology, Oncology and Tumor Immunology, Campus Virchow-Klinikum, Charité - Universitätsmedizin Berlin, Berlin, Germany

**Keywords:** autoimmune disease, autoreactivity, basophil activation test, chronic spontaneous urticaria, gain-of-function mutation, immunoglobulin E, mast cell, signal transducer and activator of transcription 3

## Abstract

**Background:**

The pathogenesis of chronic spontaneous urticaria (CSU) has not been clarified entirely. Type IIb autoimmune chronic spontaneous urticaria (CSU^aiTIIb^) is a distinct subtype of CSU that is often difficult to treat and is connected to low levels of total IgE. Previous findings indicate that an enhanced signal transducer and activator of transcription 3 (STAT3) may be responsible for reduced IgE serum levels.

**Objective:**

Our aim was to investigate a possible underlying gain-of-function mutation or activating polymorphism in *STAT3* that could be responsible for the low levels of IgE in patients with CSU^aiTIIb^.

**Methods:**

We included 10 patients with CSU^aiTIIb^ and low levels of IgE and sequenced selected single nucleotide polymorphisms (SNP) in *STAT3* associated with common autoimmune diseases. Exon sequencing was performed for the most relevant exons of *STAT3*. To test for a gain-of-function of STAT3, we performed a phospho-specific flow cytometry analysis of STAT3 in peripheral blood mononuclear cells before and after stimulation with interleukin-6.

**Results:**

No differences were found in the prevalence of the tested SNPs between our patients and a control population. Moreover, we could not find any mutations or variants on the tested exons of *STAT3*. The function of STAT3 was also not altered in our patients.

**Conclusion:**

In total, we could not find any evidence for our hypothesis that low IgE in patients with CSU^aiTIIb^ is linked to mutations in *STAT3* or altered activity of STAT3. Thus, it remains to be discovered what causes the low serum levels of IgE in patients with CSU^aiTIIb^.

## Introduction

Chronic spontaneous urticaria (CSU) is a common mast cell-driven skin disease, characterized by the occurrence of itchy wheals, recurrent angioedema, or both (1). In CSU, total immunoglobulin E (IgE) levels have been found to be elevated in a large proportion of patients (2, 3) and it is thought to play an important role in the pathogenesis of CSU. Despite this, there are some patients who present with low or very low levels of total IgE (4, 5). These patients are especially difficult to treat and are more often non-responders to treatment with Omalizumab, a therapeutic anti-IgE antibody, than those with normal or elevated total IgE (6–9). Moreover, patients with clearly defined type IIb autoimmune CSU (CSU^aiTIIb^), a subtype of CSU in which IgG autoantibodies against IgE or its high affinity receptor FcϵRI are present, express significantly lower levels of total IgE and a higher rate of other autoimmune parameters than those without it (10). It is, as of yet, unclear why this population of CSU patients exhibit such low levels of IgE and others do not.

Several findings from previous studies and observations suggest that enhanced signal transducer and activator of transcription 3 (STAT3) signaling may be responsible for the observed effects in the above-described patients. For example, a loss-of-function mutation in *STAT3* is responsible for the development of the hyper-IgE-syndrome, a condition characterized by extremely high levels of IgE and the development of recurrent infections of the skin, sinusoids and lungs (11). Interestingly, and despite the massively increased IgE levels, mast cell activation and degranulation are reduced in these patients. In contrast, gain-of-function mutations in *STAT3* are not only associated with greatly reduced IgE levels (12) but also with much higher rates of other autoimmune disorders (13–15). Moreover, Luo *et al.* investigated the JAK-STAT3 signaling pathway in CSU and found that in patients with CSU^aiTIIb^, there is a stronger expression of genes of the JAK-STAT3 signaling pathway, including STAT3 in the skin, compared to healthy subjects (16). Single nucleotide polymorphisms (SNP) in STAT3 have been found to be of relevance in autoimmune diseases (**Table 1**). Especially the incidence of thyroid autoimmune diseases is higher in patients with CSU, in particular CSU^aiTIIb^ (29).

**Table 1:** Selected single nucleotide polymorphisms in *STAT3* associated with common autoimmune diseases.

SNP	Associated autoimmune diseases
rs1053005	Grave’s disease and hashimoto thyreoditis (17)
rs3816769	Grave’s disease and hashimoto thyreoditis (18), type 1 diabetes (19)
rs6503695	Ankylosing spondylitis (20)
rs9891119	Crohn’s disease and ulcerative colitits (21)
rs744166	Crohn’s disease and ulcerative colitits (22–24), ankylosing spondylitis (20), grave’s disease and hashimoto thyreoditis (17), multiple sclerosis (25–27), psoriatic arthritis (28)
rs1026916	Crohn’s disease, ulcerative colitits (27)

Taking all this together, we hypothesized that gain-of-function mutations or activating polymorphisms in the *STAT3* gene may be present in patients with CSU^aiTIIb^ and low levels of total IgE.

## Methods

### Characterization of the patients

We included 10 patients with CSU^aiTIIb^ who had been treated at the Urticaria Reference and Excellence Center (UCARE) (30) at Chariteí - Universitätsmedizin Berlin. All patients provided oral and written informed consent that included the publication of their pseudonymized data. The study was approved by the Ethics Committee of Chariteí - Universitätsmedizin Berlin, Germany.

We selected patients with low total IgE levels and CSU^aiTIIb^. Less than 40 kU/l was chosen as a cut-off for low IgE levels, as this had previously been reported to be of significance in patients with CSU^aiTIIb^ (10, 31).

As a marker for CSU^aiTIIb^ we chose the basophil activation test (BAT), which is one of three obligatory criteria for defining CSU^aiTIIb^, as proposed by the European Academy of Allergy and Clinical Immunology (32). The BAT is positive if healthy donor basophils degranulate due to autoantibodies present in the patients’ serum. The BAT was performed as previously described (4). Briefly, the patient’s serum was thawed and incubated with fresh basophils taken from a healthy donor. Following washing and centrifugation, cells were stained with CD3, CD193 and the basophil activation markers CD63 and CD203c for measurement in flow cytometry. The BAT was considered positive, if more than 7.77% of the total basophils were both CD63 and CD203c positive. Only patients with a high proportion of double positive basophils were included in the study.

The disease activity of the patients was measured using the 7-day once-daily urticaria activity score (UAS7) (1). Additionally, patients were screened for concomitant recurrent angioedema and presence of autoimmune diseases. Among others, anti-neutrophil cytoplasmic antibodies (ANCA; considered elevated if cANCA ≥10 U/ml, pANCA ≥5 U/ml) and anti-nuclear antibodies (ANA; considered elevated if ≥1:160) were measured in patients’ serum.

### Single nucleotide polymorphism (SNP) analyses of *STAT3*

Peripheral venous blood was collected from the subjects in an EDTA tube and different SNPs in *STAT3* that were found to be of relevance in other autoimmune diseases (rs1053005, rs3816769, rs6503695, rs9891119, rs744166 and rs1026916; **Table 1**) were analyzed. Cells were lysed and genomic DNA was isolates using Gentra Puregene kit (Cat#158422, QIAGEN, Hilden, Germany) as specified by the manufacturer. PCR products were generated using the HotStarTaq Master Mix kit (Cat#20344, QIAGEN, Hilden, Germany) according to manufacturer’s instructions with the primers shown in the **Supplementary Table 1**. The thermocycler protocol was the following: 95°C for 15 min, then 35 cycles of 95°C for 30 s, 63°C for 30 s, 72°C for 30 s. Genotyping of selected SNPs was performed by Sanger sequencing (PlateSeq Service, 96w including sample clean-up, Eurofins Genomics, Ebersberg, Germany) using forward primers (**Supplementary Table 1**). A European (non-Finnish) healthy population taken from the Genome Aggregation Database (gnomAD) v2.1.1 (n=60.146) served as control group (33).

### Exon sequencing of *STAT3*

We decided to perform exon sequencing of specific exons of *STAT3* only (exons 10, 11, 13, 14, 21, 22 and 23), as most of the autoimmune-associated gain-of-function variants reported were found here (15). For the exon sequencing, blood was also taken from ten healthy volunteers as controls and to verify the quality of the samples. Genomic DNA was extracted by DNEasy Blood & Tissue Kit (Cat#69506, QIAGEN, Hilden, Germany) as specified by the manufacturer and relevant exons were amplified by PCR using Taq PCR Core Kit (Cat#201225, QIAGEN, Hilden, Germany) according to manufacturer’s instructions and using the primers listed in **Supplementary Table 2**. The thermocycler protocol was 5 min at 94°C, followed by 35 cycles of 30 s at 94°C, 30 s at 54 – 58°C (depending on primer pair), 30 s at 72°C, followed by 5 min at 72°C final elongation. Genotyping was performed by Sanger sequencing (PlateSeq Service, 96w including sample clean-up, Eurofins Genomics, Ebersberg, Germany) using forward primers (**Supplementary Table 2**).

### Analyses of functional gain-of-function of STAT3

To test functional relevance of possible gain-of-function variants of STAT3, we performed a phospho-specific analysis of STAT3 by flow cytometry before and after stimulation with interleukin-6 (IL-6). The test was performed on peripheral blood mononuclear cells (PBMC) isolated from heparinized blood of four of the patients and of four healthy controls. The PBMCs were stimulated with 20 ng/ml human IL-6 (Cat#200-06-20, PeproTech, Hamburg, Germany) for 15 min at 37°C and stored after fixation and permeabilization with formaldehyde (2%) and addition of 90% ice cold methanol. Before analysis with flow cytometry (MACS Quant, Miltenyi Biotec, Bergisch Gladbach, Germany), the cells were stained with phosphorylated STAT3 (pSTAT3) antibody (Cat#612569, Biolegend, San Diego, CA) in PBS with 1% bovine serum albumin for 30 min at room temperature. The analysis was performed using FlowJo (v.10.6.1, BD Biosciences, Franklin Lakes, NJ) and the Statistical Package for the Social Science (IBM SPSS version 27; IBM Corp, New York, NY).

## Results

Ten patients were included in the study, all of them being female with a median age of 51 years and a median disease duration of one year (**Table 2**). Median total IgE levels were 8.50 kU/l and all patients had a positive BAT, with a median of 54.1% for CD63/CD203c double positive cells. Eight out of ten patients had concomitant recurrent angioedema and six had either a diagnosed autoimmune disease or elevated levels of ANA or ANCA. The disease activity of the patients differed a lot, ranging from an UAS7 score of 3 up to 35.

**Table 2:** Patient characteristics.

Patient	Age in years	Disease duration in years	Total IgE in kU/l	CD63 and CD203c positive cells in BAT	UAS7(0-42)	Concomitant conditions
1	52	7.0	20.90	75.6%	–	Angioedema, ANA elevated (1:160)
2	60	2.0	17.00	64.3%	24	Angioedema, Hashimoto thyroiditis
3	27	8.0	10.10	44.7%*	–	Angioedema, Morbus Basedow
4	66	1.2	6.92	66.7%	14	Angioedema
5	49	1.0	6.30	21.1%	35	Angioedema, ANA elevated (1:160), pANCA elevated (7,2 U/ml)
6	72	0.8	3.25	13.6%	3	Angioedema
7	81	40.0	<2.00	82.2%	21	Angioedema, ANA elevated (1:1280)
8	26	1.0	10.80	33.0%	35	–
9	25	0.8	14.70	70.0%	–	Angioedema, Hashimoto thyroiditis
10	31	0.3	<2.00	43.9%	30	–
**Median**	**51**	**1.1**	**8.50**	**54.5%**	**24**	

*ANA, Anti-nuclear antibodies; ANCA, Anti-neutrophil cytoplasmic antibodies; BAT, Basophil activation test; IgE, Immunoglobulin E; UAS7, 7-day once-daily urticaria activity score*.

*For patient 3 the percentage of CD63 positive cells in the BAT is shown only, as no CD203c value was calculated.

The allele frequencies found for the six different SNPs in the *STAT3* gene that we analyzed showed no major differences between the patients and the control group (**Table 3**).

**Table 3:** Allele frequencies of six single nucleotide polymorphisms in *STAT3* in ten patients with CSU^aiTIIb^ compared to controls.

Single nucleotide polymorphism	rs1053005	rs3816769	rs6503695	rs9891119	rs744166	rs1026916
Single nucleotide variant	T-C	T-C	T-C	A-C	A-G	A-G
Minor allele frequency patients (n = 10)	0,25	0,30	0,35	0,30	0,35	0,70
Minor allele frequency controls (n = 60.146)	0,18	0,31	0,30	0,33	0,38	0,67

*A European (non-Finnish) healthy population taken from the Genome Aggregation Database (gnomAD) v2.1.1 (n=60.146) served as control group (33).*

Exon sequencing was performed for *STAT3* exons 10, 11, 13, 14, 21, 22 and 23. No pathogenic variants or polymorphisms were found in the ten patients. Sequencing performed on controls showed good quality in all of the probes and no differences as compared to the patients’ samples.

**Figure 1:**
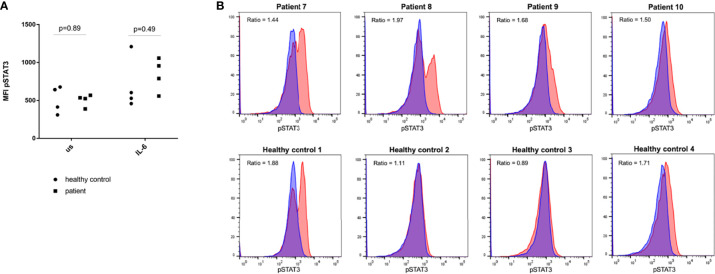
Phospho-specific flow cytometry analysis of STAT3 in peripheral blood mononuclear cells of four patients with CSU^aiTIIb^ and four healthy controls. **(A)** shows values of mean fluorescent intensity (MFI) of phosphorylated STAT3 (pSTAT3) for patients compared to healthy controls in unstimulated cells (us) and after stimulation with interleukin-6 (IL-6). For statistical comparison a Mann-Whitney-U test was performed. **(B)** shows the amount of pSTAT3 before (blue) and after (red) stimulation with IL-6 for each individual in a histogram. The ratio of the MFI of pSTAT3 before and after stimulation is indicated.

To test for an enhanced activation of STAT3, we assessed the rate of pSTAT3 after stimulation with IL-6 using phospho-specific flow cytometry. The mean fluorescent intensity (MFI) of pSTAT3 of patients and controls before and after stimulation with IL-6 is depicted in **Figure 1A**. For this, no significant differences between the groups could be found (Mann-Whitney-U, p=0.89 and p=0.49). **Figure 1B** shows the MFI for each patient and control individually and indicates the individual ratios. The mean ratio of the MFI of pSTAT3 before and after stimulation with IL-6 was 1.65 (range 1.44-1.97) in the patient group as compared to 1.40 in the control group (range 0.89-1.88) and the ratios also did not differ significantly between the two groups (Mann-Whitney-U, p=0.69).

## Discussion

In this study, we examined ten patients with CSU^aiTIIb^ and very low total levels of IgE. We hypothesized that a gain-of-function mutation or activating polymorphism in *STAT3* may be responsible for the very low levels of IgE observed in patients with CSU^aiTIIb^. However, we could not find any known pathogenic variants at the most common sites of *STAT3*. Moreover, we analyzed the most frequent SNPs in *STAT3* and found that these are not expressed exceptionally more or less compared to the control cohort. Additionally, no substantial increase of STAT3 function as analyzed by phospho-specific flow cytometry was detected. In total, we could not find enough evidence supporting our initial hypothesis.

The study has some limitations that prevent us from drawing definitive conclusions about the role of STAT3 in CSU. First, the number of patients included in the study was quite small. Especially SNP analysis should ideally be done on large patient samples in order to make a statement about the role of the different variants. Furthermore, for future experiments, it would be of interest to also include CSU patients with normal and/or elevated IgE levels and to compare for instance the amount of STAT3 expressed between distinct subtypes of CSU patients.

In addition, we sequenced only the exons of *STAT3* in which most mutations were described. Thus, although we looked at many different aspects of the *STAT3* gene and its function, including phospho-specific flow cytometry of pSTAT3, we cannot rule out that there might be other, possibly not yet discovered, mutations in some of the other exons or even the introns of this gene.

It therefore remains to be determined what the causes of the low IgE levels in CSU^aiTIIb^ patients are. Potential alternative explanations include effects on T and B cells. For example, increased frequencies of Th2 and Th17 cells have been found in lesional skin of patients with CSU (34). Th1 and Th17 mediated immune pathways, such as IFN-γ and IL-21 have been shown to decrease IgE levels (35). Thus, it can be speculated that the activity of Th17 cells in CSU is responsible for low levels of IgE in CSU. It might therefore be interesting to compare the number of Th17 cells between CSU patients with and without low IgE levels.

Another explanation for low levels of IgE in patients with CSU^aiTIIb^ could be a B-cell defect in these patients, leading to an impaired antibody class switch with imbalanced immunoglobulin production. Recent findings show that CSU patients with low IgE levels often also express lower IgA and IgG levels and higher IgM levels, which supports this hypothesis (4).

A better understanding of the diverse pathogenesis of CSU should be the aim of future studies. Especially in CSU^aiTIIb^, current treatment options are limited and knowledge about the underlying pathogenesis may help to identify appropriate and optimal therapies for each patient and to decrease the disease burden in patients with CSU.

## Data availability statement

The raw data supporting the conclusions of this article will be made available by the authors, without undue reservation.

## Ethics statement

The studies involving human participants were reviewed and approved by Ethics Committee of Chariteí - Universitätsmedizin Berlin, Germany. The patients/participants provided their written informed consent to participate in this study.

## Author contributions

MS, MMe, and KK substantially contributed to the conception and design of the study, performed analyses and interpretation of the data and drafted the manuscript. MS and NM performed the cellular assays and prepared the blood samples for the exon sequencing. TB contributed to the methodology of the genetic analyses and performed the preparation of the blood samples for the SNP analyses. JS and SF substantially contributed to the development and interpretation of laboratory tests. JS, SF, TB, and MMa provided critical input to the manuscript. All authors were involved in the final approval of the version to be published.

## Funding

This study was funded by intramural grants only. MS was supported by a medical doctoral research stipend funded by Charité and Berlin Institute of Health.

## Conflict of interest

The authors declare that the research was conducted in the absence of any commercial or financial relationships that could be construed as a potential conflict of interest.

## Publisher’s note

All claims expressed in this article are solely those of the authors and do not necessarily represent those of their affiliated organizations, or those of the publisher, the editors and the reviewers. Any product that may be evaluated in this article, or claim that may be made by its manufacturer, is not guaranteed or endorsed by the publisher.

## Abbreviations

BAT, Basophil activation test; CSU, Chronic spontaneous urticaria; CSU^aiTIIb^, Type IIb autoimmune chronic spontaneous urticaria; IgE, Immunoglobulin E; IL-6, Interleukin-6; PBMC, Peripheral blood mononuclear cells; pSTAT3, Phosphorylated signal transducer and activator of transcription; SNP, Single nucleotide polymorphism; STAT3, Signal transducer and activator of transcription 3; UAS, Urticaria activity score.
