# Supplementary material for: STAT3 gain-of-function is not responsible for low total IgE levels in patients with autoimmune chronic spontaneous urticaria
Source: Front Immunol. 2022 Jul 19;13:902652. doi: 10.3389/fimmu.2022.902652 (PMC9345496; doi:10.3389/fimmu.2022.902652)
Supplement: Supplementary file 1 [file DataSheet_1.docx]

Supplementary Material

**Supplementary table 1.** Primer sequences for *STAT3* SNP analysis.

| **SNP** | **Primer** | **Sequence (5’-3’)** |
| --- | --- | --- |
| rs1053005 | SNP1_Fw | GAGAAGCCCTGAACCCTCGC |
|  | SNP1_Rv | GCTAGCTCGCCTCTCCTGT |
| rs3816769 | SNP2_ Fw | CATGCCTGGCCTTGTCTGAGT |
|  | SNP2_ Rv | CCCGTGAGCATCATTACCTGAGA |
| rs6503695 | SNP3_Fw | CTGGCCCTTCACCAAATCAGAGT |
|  | SNP3_Rv | TCTCACAAGAACTCACTGTCTCGA |
| rs9891119 | SNP4_Fw | GCACTAGTGGATTTACAACGAGGGT |
|  | SNP4_Rv | TTGGTTGCCTTGATCACTGGGC |
| rs744166 | SNP5_Fw | ATCAGGCAGAAACATCAGAGCCA |
|  | SNP5_Rv | TGCCACAGGAACCTGGGAGGTT |
| rs1026916 | SNP6_Fw | CCCAAGGATAAGGTGCGGACTAA |
|  | SNP6_Rv | GCCCCATAGATTCCACGGACG |

**Supplementary table 2.** Primer sequences for *STAT3* exon sequencing.

| **Exon** | **Primer** | **Annealing Temperature** | **Sequence (5’-3’)** |
| --- | --- | --- | --- |
| Exon 10 | Exon1_left | 58.0 °C | CACGTGGTAGAGTGAGAGGC |
|  | Exon1_right |  | GCCACCAACTCTACCCTCAC |
| Exon 11 | Exon2_left | 55.5 °C | TATAGACAGCTTGGCCTATTTACCT |
|  | Exon2_right |  | TTTGTCCACAAAATGAAGATCTCTGA |
| Exon 13-14 | Exon3_left | 58.0 °C | GACGTTGCAGCTCTCAGAGG |
|  | Exon3_right |  | CCCCTCTCTCCCTCAAGGAA |
| Exon 21 | Exon4_left | 54.0 °C | AGTCTTTTCCCCTTCGAGGA |
|  | Exon4_right |  | GGTCAGCTTCAGGATGCTCC |
| Exon 22-23 | Exon5_left | 55.5 °C | TGAATGCGAAGTCACAGTCAGT |
|  | Exon5_right |  | AGTCTGTCAACCAAATACTCACCA |
